# The Importance of the Left Occipitotemporal Cortex in Developmental Dyslexia

**DOI:** 10.1007/s40474-018-0135-4

**Published:** 2018-01-19

**Authors:** Lisa Kronbichler, Martin Kronbichler

**Affiliations:** 1 Centre for Cognitive Neuroscience and Department of Psychology, University of Salzburg, Hellbrunnerstraße 34, Salzburg, 5020 Austria; 2 Neuroscience Institute, Christian Doppler Medical Centre, Paracelsus Medical University, Ignaz-Harrer Straße 79, Salzburg, 5020 Austria

**Keywords:** Developmental dyslexia, Occipitotemporal cortex, Visual word form area, Brain, MRI, fMRI

## Abstract

**Purpose of Review:**

Developmental dyslexia is characterized by an impaired acquisition of fluent and skilled reading ability. Numerous studies have explored the neural correlates of this neurodevelopmental disorder, with most classic accounts strongly focussing on left temporoparietal regions. We will review recent findings from structural and functional MRI studies that suggest a more important role of occipitotemporal cortex abnormalities in dyslexia.

**Recent Findings:**

Recent findings highlight the role of the occipitotemporal cortex which exhibits functional as well as structural abnormalities in dyslexic readers and in children at risk for dyslexia and suggest a more central role for the occipitotemporal cortex in the pathophysiology of dyslexia.

**Summary:**

We demonstrate the importance of the occipitotemporal cortex in for understanding impaired reading acquisition and point out how future research might enhance our understanding of functional and structural impairments in the reading network via large-scale data analysis approaches.

## Introduction

Developmental dyslexia (henceforth, dyslexia) designates impaired acquisition of reading skills which is not merely accounted for by mental age, sight defects, or insufficient schooling [1]. Affected individuals show difficulties in reading comprehension, word decoding, and recognition and similar tasks that require adequate reading skills [2, 3]. Dyslexia, which describes the difficulty in decoding the spelling and pronunciation of words, must be distinguished from reading comprehension impairments, where readers have problems understanding the meaning of what they read. Family studies show that dyslexia is heritable and has a strong genetic component [4]. Dyslexia lacks a concrete etiology and is determined via dimensional classification schemes: it describes the lower end of a continuous distribution that ranges from poor outcome to optimal outcome in word reading ability. However, in clinical practice, a rather arbitrary cutoff point is normally set to separate dyslexia from subclinical reading deficits. Reading skills are commonly related to general intelligence, although the importance of the discrepancy between reading ability and general intelligence has been refuted by recent research [5, 6].

Besides several auditory, visual, and motor dysfunction hypotheses, the prevalent (and most consistent [7]) cognitive explanatory approach for dyslexia is the *phonological deficit hypothesis*. It postulates a specific deficit to represent, access, and process speech sounds caused by inherent dysfunctions of cortical areas specialized in phonology and reading. Word reading difficulties differ somewhat across languages since their orthographies vary in the transparency of their grapheme-phoneme mappings. It is therefore easier to read Finnish or German words (transparent orthographies) as compared to English words (opaque orthography) [8], and this affects dyslexic as well as normal readers [9, 10]. Several attempts have been made to identify subtypes of dyslexia [1, 11••]. Critically, none of these typologies is universally accepted in the field of dyslexia research. Dyslexia is comorbid with other neurodevelopmental disorders like ADHD and dyscalculia and therefore it is not surprising that there is a solid correlation between reading and mathematical abilities and that these disorders might share common patterns of brain alterations [12].

In most classic accounts of brain abnormalities in dyslexia, the importance of the left temporoparietal cortex is especially highlighted, since this brain region has been closely linked with the phonological processing deficits in dyslexia [13, 14, 15•]. Although left ventral posterior occipitotemporal dysfunctions are regularly discussed, those were often seen as secondary brain deficits in dyslexia, as it was assumed that phonological processing deficits reflected in temporoparietal dysfunctions would cause disrupted development of the left occipitotemporal cortex (OTC). This account was also based on the idea that the temporoparietal cortex is especially important in the early stage of reading acquisition with phonologically based word decoding and that the OTC only emerges in later reading development. In this paper, we will review recent evidence that points to a more central role of the left OTC in dyslexia which suggests that functional and structural abnormalities of this region may constitute the most reliable and important neural correlate of developmental dyslexia. We will primarily review functional and structural MRI studies and mostly studies with alphabetic orthographies, as there are still (in our opinion) not enough studies to evaluate the consistency of neural abnormalities in dyslexia in other orthographies. One should note, however, that there are some reports showing that Chinese dyslexic readers do not show abnormalities in posterior brain regions, but rather in the middle frontal gyrus [16], although the left OTC is reliably involved in skilled reading also in logographic scripts [17]. Another study found comparable left OTC dysfunction in Chinese and English dyslexic readers [18].

## Brain Activation Abnormalities

The occipitotemporal (including the visual word form area; VWFA), temporoparietal, and left frontal regions regularly emerge in fMRI studies on dyslexia and are identified as important core regions for reading [1, 15•, 19–22]. Temporoparietal cortex (TPC) activation is often found in studies (and meta-analyses), although with some limitations. First, TPC activation seems to vary with reading proficiency in impaired and normal reading: Richlan et al. [23] identified underactivation in the left temporal areas in adult dyslexic readers whereas this effect was not replicated for children studies. Conversely, convergent temporal activation was shown for healthy infant but not for adult readers [24•]. Second, it is not yet clear how TPC abnormalities vary with orthographic depth: Although TPC underactivation in dyslexic readers was identified in deep and shallow orthographies in a recent meta-analysis [25••], this effect heavily relied on task-negative activation in dyslexic participants and group differences disappeared when deactivation relative to baseline was excluded from the analyses. Such inconsistencies appear incompatible with the prominent role the TPC is assigned to in neural models of dyslexia.

By contrast, the OCT shows convergent activation irrespective of reading proficiency as demonstrated in robust activation in child as well as adult readers, which highlights the role of the OCT not only during proficient reading but also during reading acquisition (meta-analysis [24•]). In a similar vein, child as well as adult dyslexic readers reveal convergent underactivation in the left ventral OTC (meta-analysis [23]). Abnormal activation in dyslexic readers in OTC might be unaffected by orthographic depth, since decreased neural response was identified in deep (English) as well as in shallow (e.g., Dutch, Italian) orthographies (meta-analysis [25••]). Besides consistent results on less activation in the left OTC in dyslexic readers, dyslexia is also associated with an abnormal neural response profile: Neural activation in dyslexic children is assumed to differentiate to a lesser extent between letters and other visual stimuli and between different types of words: Neural activation in OTC during the visual presentation of words as compared to false fonts is higher in children with normal reading abilities than in dyslexic children. They also showed a decreased differentiation between orthographically familiar and unfamiliar forms of real words [26]. An abnormal response profile of the OTC in dyslexia during visual word processing in terms of a decreased specialization was also found in subsequent fMRI studies, and can be considered as one of the most robust findings [27–29].

Support for the important role of the left OTC in dyslexia is also provided by training studies on children with reading disabilities, showing that systematic reading interventions (50 min per day) increased not only reading fluency but also neural response in the occipitotemporal cortex [30]. This effect was not replicated for “communal interventions” (i.e., interventions often provided within school settings). Another study on dyslexic readers investigated the effects of training in the domains of phonology, attention, or visual word recognition on neural response [31•]. The authors found that, irrespective of training type, significant increase in activation was only found in occipitotemporal regions, which (once more) highlights the importance of the OTC in literacy skills. ERP assessments in prereaders could show that occipitotemporal sites exhibit a delayed N1 component in infants who develop dyslexia later on in development [32]. In a similar vein, print knowledge was identified as a reliable predictor of later reading performance as early as reception class level at the ages of ~ 4 and 6 [33]. At the neuronal level, fMRI assessments during orthographic processing (letter vs. false font) in prereaders revealed a decreased specialization for letters in posterior dorsal regions [34]. Similar to findings of the training study of Shaywitz et al. [30] mentioned above, abnormal neural activation was improvable by supplementary reading intervention. Additional support for abnormal posterior brain activation prior to reading onset was provided by Raschle et al. [35]: They examined neural activation in kindergarten children during a sound matching task and found reduced activation in the OTC and TPC in infants with a family history of dyslexia.

Taken together, developmental dyslexia is frequently associated with a decreased specialization for letters and words in the OTC. Decreased neural response during reading-related processes in dyslexia seems to be invariant to orthographic depth and is robustly identified in adult as well as infant reading. Altered activation is also found in prereading studies suggesting early developmental brain alterations prior to reading acquisition [36•]. Notably, although reading abilities are often defined in relation to general intelligence, neuroimaging studies revealed evidence for the contrary: Tanaka et al. [37] assessed phonological processing in poor readers with either high or low IQ and found that both groups exhibited similar patterns of reduced neural activation in the OTC and TPC. With respect to the relationship between reading comprehension and word reading, one fMRI study provided evidence that decreased activation of the left OTC during reading is specifically associated with problems in word reading and not with impaired reading comprehension [38]. In a very recent study, Perrachione et al. [39••] found diminished neural adaptation in dyslexic readers compared to that in normal readers while processing written words in the OTC. Neural adaptation is assumed to reflect an efficient processing of sensory input and it is defined as the decreased blood oxygen level dependent (BOLD) response towards a repeatedly presented stimulus in regions critically involved in the processing of the investigated stimulus category. Strikingly, diminished neural adaptation was not restricted to reading-specific material but was also identified during visual object and face perception. Assessment of functional MRI during visual processing found further support for the notion that deficient activation in dyslexia may not be restricted to reading material but rather reflects a more general impairment of the OTC: Dyslexic readers exhibited decreased OTC activation not only for word-like stimuli but also for numbers and abstract symbol strings [40•]. Decreased activation for non-word stimuli was also found in an earlier study that reported diminished activation not only in the left occipitotemporal word-selective regions for visual words but also in the right fusiform face area in response to visual faces in dyslexic children [41]. Collectively, these recent findings suggest that abnormal left OTC function in dyslexia is not restricted to visual word processing and may therefore not be solely caused by a lack of reading experience in dyslexic readers, as also suggested by other fMRI findings reviewed in this section.

## Structural Brain Abnormalities

First neuroanatomical assessments of structural brain abnormalities in dyslexia arose from post-mortem investigations [42]. Abnormalities included a reduced leftward asymmetry of the planum temporale and ectopias located in the perisylvian regions. With the beginning of neuroimaging in living individuals, voxel-based morphometry (VBM) assessments showed gray matter abnormalities in the ventral OTC [43, 44], although the direction of altered findings was rather inconsistent. Furthermore, such gray matter abnormalities could not be replicated thoroughly [45]. On the contrary, gray matter reduction in dyslexic readers in OTC was identified in a meta-analysis by Linkersdörfer et al. [46]: The authors conducted a meta-analysis on VBM studies which revealed that abnormal neural activation and gray matter alterations in dyslexia overlapped in the left fusiform gyrus. In a similar fashion, Altarelli et al. [47] examined cortical thickness of dyslexic children around their individual peak of functional activation towards visually presented words. Dyslexic children showed decreased cortical thickness in word-selective OTC but not in other cortical areas. Notably, this effect was mainly driven by differences in healthy vs. impaired female readers. Decreased cortical thickness (and increased gyrification) in the OTC is also shown by a very recent study of Williams et al. [48]. There is also evidence that gray matter abnormalities in the OTC are specific for dyslexia and not found in readers with a specific reading comprehension impairment but no deficit in word reading per se [49].

Similar to functional MRI investigations, structural assessments show variations in the neuroanatomy of reading-related regions in at-risk prereaders. Structural alterations prior to reading acquisition include atypically small sulcal basins [50•] and reduced gray matter volume in the OTC as well as TPC [51, 52]. Here, gray matter alterations were associated with a delay in language acquisition [52] and a family history of developmental dyslexia [51, 52]. Volume indices also correlated positively with rapid automatized naming tasks in these cortical areas [51].

Genetic imaging studies have also demonstrated an association between OTC structure and genetic markers of dyslexia [4]. Skeide et al. [53•] investigated single-nucleotide polymorphisms (variations in certain DNA sequences) often associated with literacy performance in 19 genes in kindergarten schoolchildren (prereaders; 5–6 years). The *NRSN1* gene was related to gray matter volume in the VWFA. The genetically associated volume profile of the VWFA (gVWFA) was able to classify readers into dyslexic and control individuals. Furthermore, gVWFA accurately distinguished between later dyslexic and normal reading skills in the prereader sample. In another recent genetic imaging study, a link was found between a genetic risk factor for dyslexia and dyscalculia and decreased volume and brain activation of the fusiform gyrus in the left OTC [54].

Despite the large number of studies pointing to the importance of structural abnormalities in the OTC for dyslexia, it should be noted that the literature on structural brain abnormalities in dyslexia is plagued by inconsistencies and failures to replicate (for an excellent recent critical review, see [11••]). These inconsistencies are also reflected in a large-scale study of structural abnormalities based on a multi-site dyslexia dataset collected via the Dyslexia Data Consortium. This analysis showed that most differences in gray and white matter volume disappeared when the analysis adjusted for differences in whole brain volume and that dyslexia was associated with more heterogeneity of gray matter volume in most regions of the brain [55••].

## Structural Brain Connectivity

Dyslexia was early conceptualized as a disconnection syndrome [56], consistent with the explanation that impaired reading might be closely associated with impaired connectivity and disturbed white matter tracts between reading-related brain areas. Accordingly, numerous studies have explored structural connectivity in dyslexia using diffusion-weighted MRI [57]. As in studies exploring functional MRI and structural MRI, early assessments mainly focused on connectivity involving the left TPC, including the arcuate fasciculus and the corona radiata, because of their assumed link with phonological processing. A number of findings from these studies indicated abnormalities in white matter tracts but with considerable inconsistency on which track is specifically involved [11••]. Regarding the OTC, recent studies provided some evidence for white matter abnormalities. For example, familial risk for dyslexia in prereading children was mainly associated with deficits in a left ventral white matter tract, the left inferior fronto-occipital fasciculus [58•], and integrity of this ventral tract was related to both familial risk for dyslexia and later reading ability in a longitudinal DWI study [59]. Interestingly, integrity of this ventral pathway was found to be associated with performance on an orthographic processing task [60]. However, there are also inconsistent results from studies indicating no association between integrity of ventral white matter tracts and risk for dyslexia in prereaders, but rather highlight an association between dorsal tracts and dyslexia risk [61]. In summary, there is inconsistent evidence for abnormalities of ventral white matter tracts in the OTC in dyslexia and reading impairment. Generally, although there is a consensus on the importance of white matter tract abnormalities in dyslexia, there is still no clear consensus on which specific white matter abnormalities are reliably associated with dyslexia. It should be noted that DWI studies can differ in numerous aspects [11••], which makes formal meta-analysis across studies difficult and may partly explain the lack of a clear consensus.

## Functional Brain Connectivity

To date, relatively few fMRI studies have reported findings on impaired functional brain connectivity in dyslexia, despite the clear interest on disordered communication between brain regions. With respect to the left OTC, we have recently shown that this region reveals less connectivity with the left inferior frontal regions [62••]. Importantly, this reduction of functional connectivity of the OTC was not merely found during two different reading tasks but also during the task-free resting state, thus highlighting a general and permanent disruption of the left OTC in dyslexia. Comparable results were reported in other fMRI connectivity studies during reading tasks [29, 63, 64]. One recent resting fMRI connectivity study with Chinese dyslexic children also reported decreased connectivity of the left OTC with the left frontal regions [65]. Another study reported widespread functional connectivity abnormalities in dyslexia using whole-brain data-driven analysis approach. They also identified reduced coupling between the ventral visual regions in the OTC and reduced coupling between the visual and prefrontal regions [66]. However, some studies did not identify impaired functional connectivity involving the left OTC but revealed other abnormalities in dyslexia during reading or rest [67–70]. Compared to other brain disorders, there are relatively few fMRI connectivity and especially resting state connectivity studies on dyslexia. Even fewer studies employ effective connectivity techniques like Dynamic Causal Modelling (DCM), which would allow for a more targeted analysis of connectivity abnormalities and the role of top-down and bottom-up connectivity in dyslexia and which turned out promising in understanding connectivity during visual word processing [71]. The small number of DCM studies in dyslexia does not yet show a consistent pattern for the OTC (or other regions of the reading network) [72, 73•]. Clearly, more and larger studies are needed to characterize potential abnormalities in connectivity of the OTC in dyslexia more reliably, also because studies in non-impaired readers suggest that the role of the OTC and its emergence during development is strongly determined by structural and functional connectivity [74, 75].

## Functions of the OTC in Non-Impaired Reading

The OTC is consistently identified in studies assessing literacy skills in normal and dyslexic reading. This area is assumed to accommodate a functional region—VWFA—which computes representations of visually presented words irrespective of variances in size, font, location, and case [76]. Initial accounts of the VWFA assumed that it is mainly responsible for prelexical orthographic processing and does not respond to auditory words [77]. However, recent neuroimaging findings account this of the VWFA: For example, a number of findings now strongly argue for orthographic (whole word) representations in the sense of an orthographic lexicon within the VWFA [78, 79]. Furthermore, VWFA seems to be involved in accessing the orthography of auditorily presented words [80]. Assuming that abnormal VWFA activation is also related to disturbed auditory processes, this might be an interesting link to spelling deficits identified in some dyslexics or the finding that lesions in the VWFA are often accompanied by acquired alexia.

Variable response properties suggest that VWFA activation also contributes to other functions besides the mere visual representation of words. In their “The interactive account of the ventral occipitotemporal contributions to reading,” Price and Devlin [81] propose that the function of this region varies depending on its interaction with other areas. Therefore, top-down input from auditory processes or lexical information might alter VWFA activation for efficient stimulus processing. An alternative approach is that there exist two functionally different regions within the OTC [82, 83]: One that is relevant for the graphemic description of a word, irrespective of its location, font, or size. This region corresponds to what is often termed *VWFA* [82]. The other region is located lateral to the left of the VWFA and is engaged in a variety of lexical tasks and responds to written *and* spoken words [82, 83]. It is therefore assumed to be modality independent and to link semantics to lexical representation for written or oral output. Although there is some support for this approach, this functional distinction needs future investigations since—due to their spatial proximity—these two regions are confounded in previous studies and task-dependent individual localizations of the VWFA complicate a retrospective separation even more.

## Conclusions

In summary, the OTC is most consistently identified in infant and adult readers irrespective of orthographic depth and is even shown in fMRI assessments of non-alphabetic writing systems. Dyslexic readers not merely show decreased neural response in the OTC but rather indicate unspecific activation towards a reading material. Although less consistent, several structural MRI assessments highlight the role of structural alterations in the OTC in dyslexic readers. Strikingly, structural as well as functional alterations become evident even before reading acquisition in preschool children and are able to accurately predict later reading disabilities. The central importance of the OTC in reading and reading-related skills like spelling and (rapid) object naming might be one potential explanation for the relatively broad literacy impairments of dyslexia. Connectivity impairments between the OTC and frontal areas might be indicative of a disrupted linkage between orthographic and phonological word representations what in turn leads to inefficient and slow reading performance.

Considering the evidence of abnormalities in dyslexia in various research domains ranging from behavioral RT measures to functional brain activation and even genetic factors, it seems unlikely that there is a single mechanistic explanation for dyslexia. Additionally, there is an ongoing discussion about whether there are qualitative differences in the neuronal mechanisms that underlie dyslexia, meaning that dyslexic symptoms might be based upon slightly diverging “neuronal subtypes” of dyslexia. The assumption of different neuronal subtypes might account for the sometimes inconsistent findings produced in this field. To illustrate, it remains up to future studies to show how occipitotemporal abnormalities relate to temporoparietal and frontal activation patterns and why certain tasks elicit decreased neural response in dyslexic readers whereas others do not. Furthermore, alterations in functional activation and structure might also be influenced by comorbid disorders like ADHD and dyscalculia and subclinical traits; at the moment, there is not much brain data directly exploring the relationship between dyslexia and other neurodevelopmental disorders and between reading ability and other skills and traits.

Advanced approaches might provide suitable methods to classify dyslexic subgroups in a data-driven manner based on brain function, connectivity, and structure: Such methods are already successfully applied in other psychiatric disorders [84]. Besides, advanced developments in the field of computational neuroanatomy and quantitative MRI might also be of great relevance for an improved and more sensitive analysis of brain structure [85]. In general, there is an urgent need for a careful integration of large-scale functional and structural MRI datasets on dyslexia and increased data sharing. The first steps and promising results have already been provided by structural MRI data collected by the Dyslexia Data Consortium [55••]. But, large-scale, publicly available datasets are also needed for functional MRI data; such data sharing already had a large impact on research on the brain basis of another neurodevelopmental disorder, for example, in the field of autism research [86].
